# Pilot research on predicting the sub-volume with high risk of tumor recurrence inside peritumoral edema using the ratio-maxiADC/meanADC from the advanced MRI

**DOI:** 10.1007/s12672-025-03199-3

**Published:** 2025-09-24

**Authors:** Jinling Zhang, Haobin Liu, Yuxiao Wu, Jian Zhu, Yuting Wang, Yijun Zhou, Mingguang Wang, Quanyu Sun, Fengyuan Che, Baosheng Li

**Affiliations:** 1 Cancer Center, Linyi People`s Hospital, Shandong Second Medical University, Linyi, 276000 China; 2 School of Clinical Medicine, Shandong Second Medical University, Weifang, 261053 China; 3 Department of Medical Oncology, Shandong Cancer Hospital and Institute, Shandong First Medical University, Shandong Academy of Medical Science, Jinan, 250117 China; 4 Department of Radiology, Sichuan Provincial People’s Hospital, University of Electronic Science and Technology of China, Chengdu, 610072 China; 5 Department of Neurosurgery, LinyiPeoplès Hospital, Shandong Second Medical University, Linyi, 276000 China; 6 Department of Radiology, LinyiPeoplès Hospital, Shandong Second Medical University, Linyi, 276000 China; 7 Department of Neurology, LinyiPeoplès Hospital, Shandong Second Medical University, Linyi, 276000 China; 8 Key Laboratory of Cancer Prevention and Therapy, Tianjin Medical University Cancer Institute and Hospital, National Clinical Research Center for Cancer, Tianjin’s Clinical Research Center for Cancer, Tianjin, 300060 China; 9 Department of Radiation Oncology, Tianjin Medical University, Tianjin, 300060 China

**Keywords:** Tumor recurrence, Peritumoral edema, Glioblastoma, ADC map

## Abstract

**Purpose:**

This study aimed to identify key image parameters from the traditional and advanced MR sequences within the peritumoral edema in glioblastoma, which could predict the sub-volume with high risk of tumor recurrence.

**Materials and methods:**

The retrospective cohort involved 32 cases with recurrent glioblastoma, while the retrospective validation cohort consisted of 5 cases. The volume of interest (VOI) including tumor and edema were manually contoured on each MR sequence. Rigid registration was performed between sequences before and after tumor recurrence. The edema before tumor recurrence was divided into the subedema-rec and subedema-no-rec depending on whether tumors occurred after registration. The histogram parameters of VOI on each sequence were collected and statistically analyzed. Beside Spearman’s rank correlation analysis, Wilcoxon’s paired test, least absolute shrinkage and selection operator (LASSO) analysis, and a forward stepwise logistic regression model(FSLRM) comparing with two machine learning models was developed to distinguish the subedema-rec and subedema-no-rec. The efficiency and applicability of the model was evaluated using receiver operating characteristic (ROC) curve analysis, image prediction and pathological detection.

**Result:**

Differences of the characteristics from the ADC map between the subedema-rec and subedema-no-rec were identified, which included the standard deviation of the mean ADC value (stdmeanADC), the maximum ADC value (maxiADC), the minimum ADC value (miniADC), the Ratio-maxiADC/meanADC (maxiADC divided by the meanADC), and the kurtosis coefficient of the ADC value (all *P* < 0.05). FSLRM showed that the area under the ROC curve (AUC) of a single-parameter model based on Ratio-maxiADC/meanADC (0.823) was higher than that of the support vector machine (0.813) and random forest models (0.592), compared to the retrospective validation cohort’s AUC of 0.776. The location prediction in image revealed that tumor recurrent mostly in the area with Ratio-maxiADC/meanADC less than 2.408. Pathological detection in 10 patients confirmed that the tumor cell dotted within the subedema-rec while not in the subedema-no-rec.

**Conclusion:**

The Ratio-maxiADC/meanADC is useful in predicting location of the subedema-rec.

**Supplementary Information:**

The online version contains supplementary material available at 10.1007/s12672-025-03199-3.

Glioblastoma (GBM) is a common malignant intracranial tumor, which is classified into high-grade and low-grade gliomas based on malignancy extent by pathologist. High-grade gliomas are often accompanied by extensive peritumoral edema due to their significant invasiveness [1]. The mechanisms of peritumoral edema formation in high-grade glioma is complicated. In addition to blood flow obstruction and increased vascular permeability caused by tumor micro-infiltration or inflammatory factors, other mechanisms of peritumoral edema formation remain unclear [2]. The extent of tumor micro-infiltration within the peritumoral edema is difficult to evaluate, which poses a huge challenge in determining surgical margins and postoperative radiotherapy target boundary [3]. Complete resection of glioblastoma and adjuvant radiotherapy are both identified as indenpendently prognostic factors. Furthermore, it is controversial between National Comprehensive Cancer Network (NCCN) and European Society for Medical Oncology (ESMO) guidelines regarding the extent of peritumoral edema around glioblastoma as to which part of it should be encompassed by adjuvant radiotherapy treatment [4, 5]. The reason for the controversy is that it is difficult to delineate the boundaries of tumor micro-infiltration within the peritumoral edema using traditional CT or MR images.

Recent researches have focused on exploring the micro-infiltration area within the edema around tumor lesions in high-grade glioma through advanced imaging techniques. PET-CT shows promise in differentiating the micro-infiltration area within perilesional edema by means of the standardized uptake value (SUV) [6]. Meanwhile, research reveals that magnetic resonance spectroscopy (MRS, a functional technique) has a better ability to differentiate brain gliomas from nonneoplastic lesions using the Cho/Cr ratio than other methods [7].Perfusion-weighted imaging (PWI, functional MRI), consisting of T2*-based Dynamic Susceptibility Contrast (DSC) and T1-based Dynamic Contrast Enhancement (DCE), has been explored to assess the micro-infiltration around pathological lesions in glioma depending on parameters such as Cerebral Blood Volume (CBV), Cerebral Blood Flow (CBF), Ktrans (volume transfer constant), Vp (plasma volume), and Kep (rate constant). Diffusion-weighted imaging (DWI, a microstructural imaging modality), diffusion kurtosis imaging (DKI), and diffusion tensor imaging (DTI) use the apparent diffusion coefficient (ADC) value, fractional anisotropy (FA) value, and mean kurtosis (MK) values, respectively, to discriminate between micro-infiltrative and non-infiltrative areas around glioma [8, 9].

It is widely accepted that the ADC value is negatively correlated with the degree of tumor malignancy, which is because the movement ability of free water molecules varies due to the restriction imposed by tumor cells. At the same time, research confirmed that the extent of vasogenic edema usually positively correlates with the ADC value [10]. As the above research has revealed that tumor recurrence is related to this micro-infiltration within the perilesional edema in high-grade glioma. In the same time, considering the significant difference of the influence from the vasogenic edema and micro-infiltrative tumor on the ADC value, we hypothesize that some parameters from ADC map could differentiate the sub-volume of the edema surrounding glioblastoma with a high risk of recurrence(subedema-rec) and the sub-volume with a low risk of recurrence(subedema-no-rec) effectively. The objective of this study is to develop an image parameter model from the ADC map. This will be achieved by comparing the traditional logistic regression model with machine learning models, with the aim of creating a model competent enough to discriminate between subedema-rec and subedema-no-rec.

## Materials and methods

### Patients

In this study, the medical records of fifty patients with GBM were retrospectively reviewed. These patients were treated at Linyi People’s Hospital affiliated to Shandong Second Medical University from March 2013 to March 2016. An additional retrospective cohort of 5 patients was used as a validation group. These patients were treated at Linyi People’s Hospital affiliated to Shandong Second Medical University from January 2022 to January 2024. The inclusion criterion were shown as follows: identified as GBM by a pathologist; macroscopic edema around the tumor lesion in the MR image; recurrence lesions were confirmed to be partly or wholly within the edema area identified on the initial MR image; received an enhanced MR scan (including diffusion-weighted MRI) every 3 months regularly. This study was approved by the institutional Research Ethics Board (REB) of Linyi People’s Hospital, and the requirement for ethical approval documentation was waived for both the retrospective cohort and the retrospective validation cohort. All the patients received concurrent chemo-radiotherapy (CCRT) with temozolomide (TMZ) and adjuvant TMZ after maximal safe tumor resection.

### MR-DWI parameters

MR scan includes enhanced T1-weighted images (WI), T2-weighted FLAIR images and the DWI image acquired with a 3.0 T MRI scanner (Siemens Magnetom Verio; Erlangen, Germany). Scan parameters of enhanced T1 were: TR = 2000 ms, TE = 20.0 ms; for T2-FLAIR: TR = 8500 ms, TE = 90 ms. Both sequences shared identical spatial resolution: slice thickness = 4.0 mm, slice spacing = 0.5 mm, FOV = 220 × 220 mm, matrix size = 512 × 512 (in-plane resolution 0.43 × 0.43 mm²), and number of excitations = 1. Using the contrast agent Gd-DTPA (concentration is 469 mg/ml), the dose was 0.1 mmol/kg. It was injected through the upper arm vein by a high-pressure syringe. The injection flow rate was 2.5 ml/s. Injection was performed at the beginning of the first phase scan of dynamic enhanced scanning. Then, post-enhanced transverse, coronal, and oblique sagittal scans of T1WI were performed together with the transverse section and a fat suppression sequence.

The parameters of DWI used in this imaging protocol were as follows: breath-hold DW echo planar images were performed with a matrix size of 128 × 128; slice thickness of 5 mm; b-value of 0 and 1000 s/mm²; TE range: 95; TR: 5900; receive bandwidth: 62.5 kHz; NEX: 2; FOV: 22 cm × 22 cm, DWI images were resampled to match T1w resolution (0.43 × 0.43 × 4.0 mm³) using linear interpolation during rigid registration in 3D Slicer.

### Image analysis and parameters collection

The patient demographics, consisted of the parameter from enhanced T1WI, T2-weighted FLAIR images and the DWI image from the beginning of diagnosis to the last follow-up after tumor resection and the information of adjuvant chemoradiotherapy were reviewed. All images were import into the free and open-source medical image analysis software 3D Slicer.

ADC maps before and after tumor recurrence were produced by Siemens work station from DWI images. The enhanced T1WI before and after tumor recurrence, together with T2-weighted FLAIR images and the ADC map before tumor recurrence, were registered using the quantification modules of software 3D Slicer by rigid registration.

Tumor lesions before and after tumor recurrence were contoured on enhanced T1WI, and edema volume before tumor recurrence was contoured on T2-weighted FLAIR images by two radiation oncologists with around 10 years of experience in neuroimaging.

After registration, the subedema-no-rec was defined as the volume of the result that the edema volume before tumor recurrence subtracted off the tumor volume after tumor recurrence. The subedema-rec was the remaining part of edema before tumor recurrence except the subedema-no-rec. Recurrent tumor regions were delineated on contrast-enhanced T1-weighted images (T1w) at follow-up, and subedema-rec was defined as the overlapping volume between pre-recurrence edema (on initial T2-FLAIR) and recurrent tumor contours (on follow-up T1w) after rigid registration.

The following parameters of subedema-rec, subedema-no-rec, and tumor volume before and after tumor recurrence in the enhanced T1WI and ADC map were collected from the software 3D Slicer: the mean ADC value (meanADC), the standard deviation of the mean ADC value (StdmeanADC), the maximum ADC value (maxiADC), the minimum ADC value (miniADC), the median ADC value (medianADC), the skewness of the ADC value (skewnessADC), and the kurtosis of the ADC value (kurtosisADC). The corresponding parameters on enhanced T1WI were also collected. The ratio of the maxiADC of the region of interest (VOI) to the meanADC value of VOI was named the Ratio-maxiADC/meanADC.

### Image prediction of lesion location

On the Python 3.1.2 platform, the protocol as follow was proceeded. After defining the path of the target image, which was the slice with the largest tumor volume accompanied by peritumoral edema, automatic image segmentation was set up in units of 2 × 2 voxel tiles, 2.5 × 2.5 voxel tiles and 3 × 3 voxel tiles, respectively. Heat map was generated based on the result of Ratio-maxiADC/meanADC in above units. The heat map was automatically stored in the directory of the same path as the target image and stored as a JPG file. The commands were listed in supplementary file 1.

### Pathological detection

Preoperatively, after cases was diagnosed as high-grade gliomas based on a comprehensive analysis of patient symptoms, signs, and imaging examinations such as MRI, the planned puncture points of this case were set at the positions which located in the peritumoral edema uniformly. The planned puncture points were proceeded by multi-points biopsy guided by the surgical navigation system stored with the puncture plan. When the operation was proceeded, after the dura mater was opened, punctures were carried out according to the planned puncture points indicated by the navigation system. The puncture numbers were serially marked. Subsequently, the specimens were sent for fixation in glutaraldehyde. Thereafter, a series of processes including dehydration, clearing, and paraffin infiltration were carried out. Following paraffin embedding, the specimens were sectioned to a thickness of 0.1 mm and then subjected to hematoxylin-eosin (HE) staining. Subsequently, observation was conducted under magnifications of 10 × 10 and 10 × 40. By means of pathological identification, cases other than glioblastoma were excluded. Finally, the typical images corresponding to each planned puncture point were stored and recorded in accordance with the case number.

### Patient follow-up

Patients came back to hospital and were followed up every 3 months until death or the last follow-up examination. Patients who could not return to our hospital regularly were followed up by phone or through apps. All patients received enhanced T1WI, T2-weighted FLAIR images and the DWI image scans at each follow-up point, and the 2010 McDonald’s criteria was used to evaluate the local control of the tumor.

### Statistical analysis

Spearman’s rank correlation analysis was employed to explore the relation between the image parameters of VOI and volume change of the tumor or edema. Wilcoxon’s paired test was used to identify the difference of the histogram characteristics of the sub-volume inside edema. Least absolute shrinkage and selection operator (LASSO) analysis was used for preliminary investigation to select parameters for avoiding collinearity. The pre-selected parameters were further assessed via a forward stepwise logistic regression model (FSLRM) and two machine learning model, specifically the support vector machine (SVM) and random forest (RF) models, for the purpose of distinguishing between subedema-rec and subedema-no-rec. The receiver operating characteristic curve (ROC) was used to investigate the efficiency of the logistic regression model. P value of less than 0.05 was considered to indicate a significant difference. All analyses were performed using STATA/MP statistical software (Stata software version 18.0; Stata Corp., College Station, TX, USA).

## Result

### Patients characteristics

**Fig. 1:**
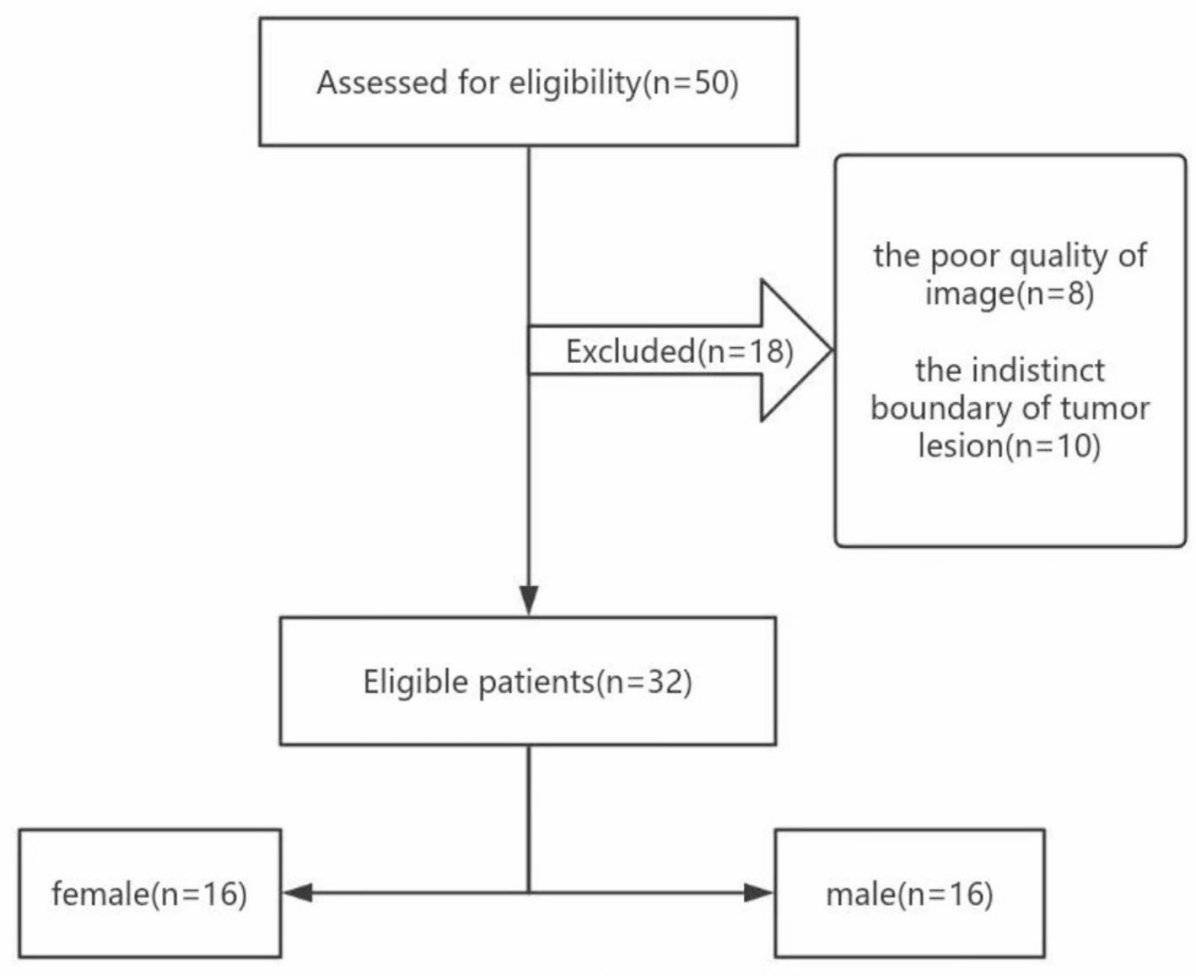
the flowchart of patient collection in the retrospective cohort

The Medical data from 16 male and 16 female patients were retrospectively analyzed, after excluding 8 patients for poor image quality and 10 patients for indistinct tumor boundaries. (Fig. 1). The mean age of the patients was 50.6 years. The mean time interval of tumor recurrence determined by MR scans was 2.3 months (range: 1–6.7 months). The mean volume of the lesion as tumor recurrent increased from 2353 ± 253 mm³ to 5879 ± 266 mm³, while the mean volume of peritumoral edema also increased from 2353 ± 253 mm³ to 5879 ± 266 mm³. In the retrospective validation cohort of 5 patients, the mean age of the patients was 40.2 years and the meantime interval of tumor recurrence was 5.3 months. The mean volume of the tumor increased from 4178 (from 2082 to 18285) mm³ to 21,212 (from 15740 to 45007) mm³ and the mean volume of peritumoral edema increased from 12,120 (from 9728 to 15152) mm³ to 10,524 (from 6701 to 70347) mm³ as tumor recurrent. The clinical information of patients was listed in Table 1.

**Table 1:** Summary of patients in this study

Case	Sex	Age	kps		Resection type	Chemoradiotherapy or not
1	M	56	90		Totally resected	N
2	F	42	90		Totally resected	N
3	M	73	90		Subtotal resection	Y
4	F	48	90		Totally resected	N
5	F	54	90		Totally resected	Y
6	M	62	80		Totally resected	N
7	M	41	80		Totally resected	Y
8	F	44	90		Totally resected	N
9	F	27	90		Totally resected	N
10	M	51	90		Totally resected	Y
11	M	72	90		Totally resected	Y
12	M	54	80		totally resected	N
13	M	42	80		Subtotal resection	Y
14	M	66	80		Totally resected	N
15	M	33	90		Totally resected	Y
16	F	25	80		Totally resected	N
17	F	52	80		Totally resected	Y
18	F	70	80		Totally resected	Y
19	M	33	80		Totally resected	N
20	F	76	70		Totally resected	Y
21	F	46	80		Totally resected	N
22	M	37	80		Totally resected	N
23	F	52	90		Totally resected	Y
24	M	26	80		Subtotal resection	Y
25	M	34	60		Totally resected	Y
26	F	49	70		Totally resected	N
27	M	52	60		Subtotal resection	Y
28	F	73	80		Totally resected	Y
29	M	64	80		Totally resected	Y
30	F	67	60		Totally resected	Y
31	F	46	70		Totally resected	Y
32	F	51	65		Totally resected	Y
F: female, M: male. Y: yes, N: no.						

### The change of image parameters of tumor as tumor recurrence

**Fig. 2:**
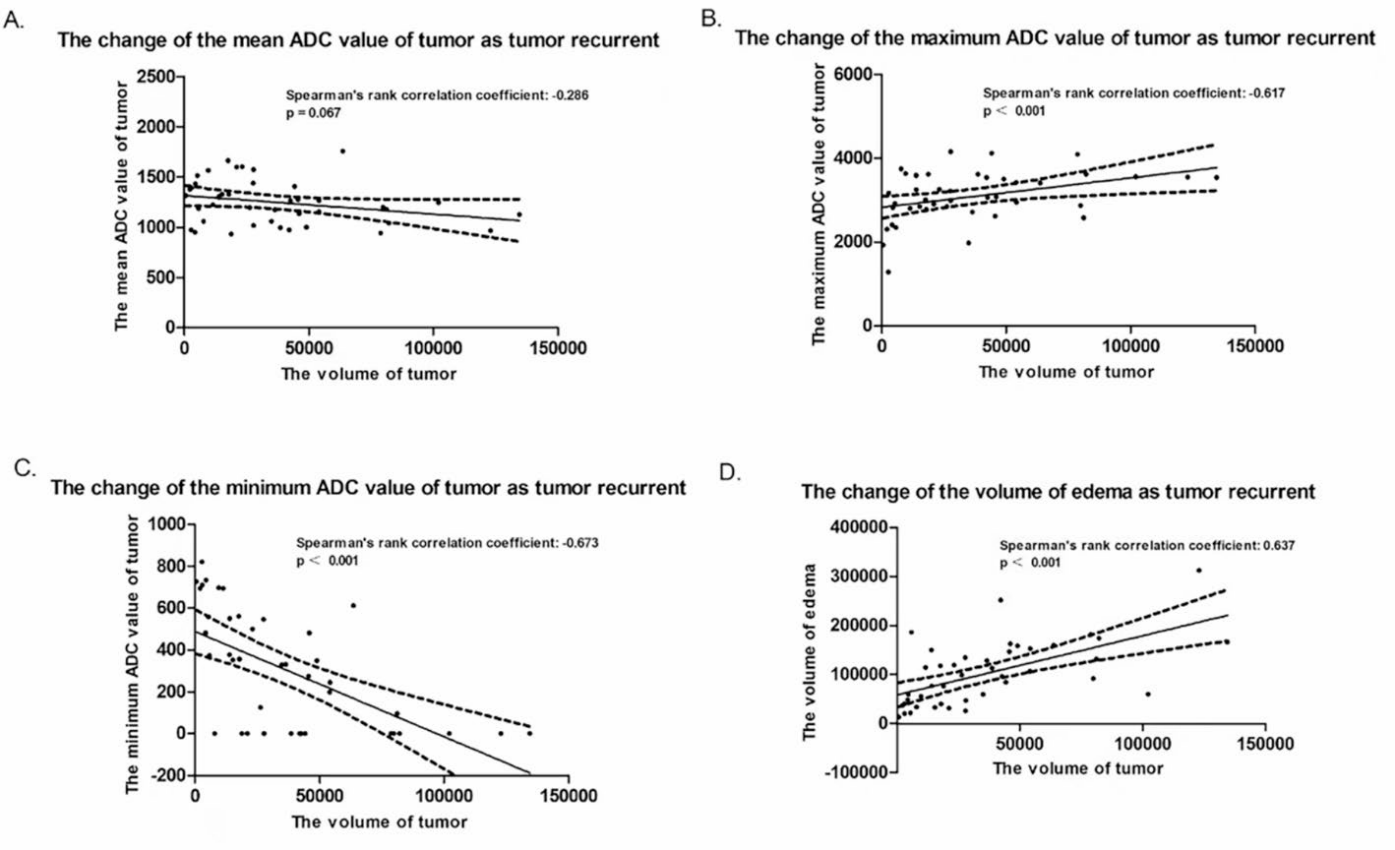
The change of image parameters on tumor as tumor recurrence. As tumor recurrent, the meanADC of tumor (A), the maxiADC of tumor (B)and the volume of peritumoral edema all increased (D), while the miniADC of tumor (C) decreased

In the retrospective cohort, the meanADC of the tumor decreased as the tumor volume increased, but the statistical relationship between them was not significant (Spearman’s ρ= -0.286, *P* = 0.067). The maxiADC increased as the tumor volume increased, and the relationship between them was statistically significant (Spearman’s ρ= -0.617, *P* = 0.001). The significant relationship between the miniADC of the tumor and tumor volume, as well as the significant relationship between the peritumoral edema volume and tumor volume during GBM recurrence were both confirmed by the analysis of the Spearman correlation method (Spearman’s ρ= -0.672, *P* = 0.001; Spearman’s ρ = 0.637, *P* = 0.001) (Fig. 2).

### The investigation on the difference of image parameters among sub-volumes of peri-tumoral edema

**Fig. 3:**
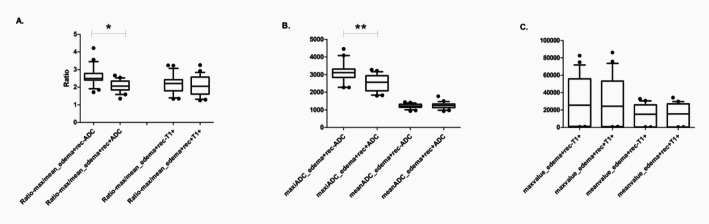
The difference of image parameters among sub-volumes of peri-tumoral edema. (A) The difference of Ratio-maxiADC/meanADC between sub-volumes within edema in ADC map and enhanced T1 sequence. (B) The difference of maxiADC and meanADC between sub-volumes within edema. (C) The difference of max value and mean value between sub-volumes within edema in enhanced T1 sequence

No significant difference of image parameters among the sub-volumes of peritumoral edema on enhanced T1WI was confirmed (all *P* > 0.05). For the parameters from the ADC map, the meanADC, medianADC, stdmeanADC, kurtosisADC, and skewnessADC had no significant difference among the sub-volumes of peritumoral edema either (all *P* > 0.05). However, significant differences of the maxiADC, miniADC and Ratio-maxiADC/meanADC were confirmed among the sub-volumes of peritumoral edema (all *P* < 0.05). The above result was partly shown in Fig. 3.

### The investigation of the efficiency of the parameter on differentiating the sub-volume of peri-tumor edema

**Fig. 4:**
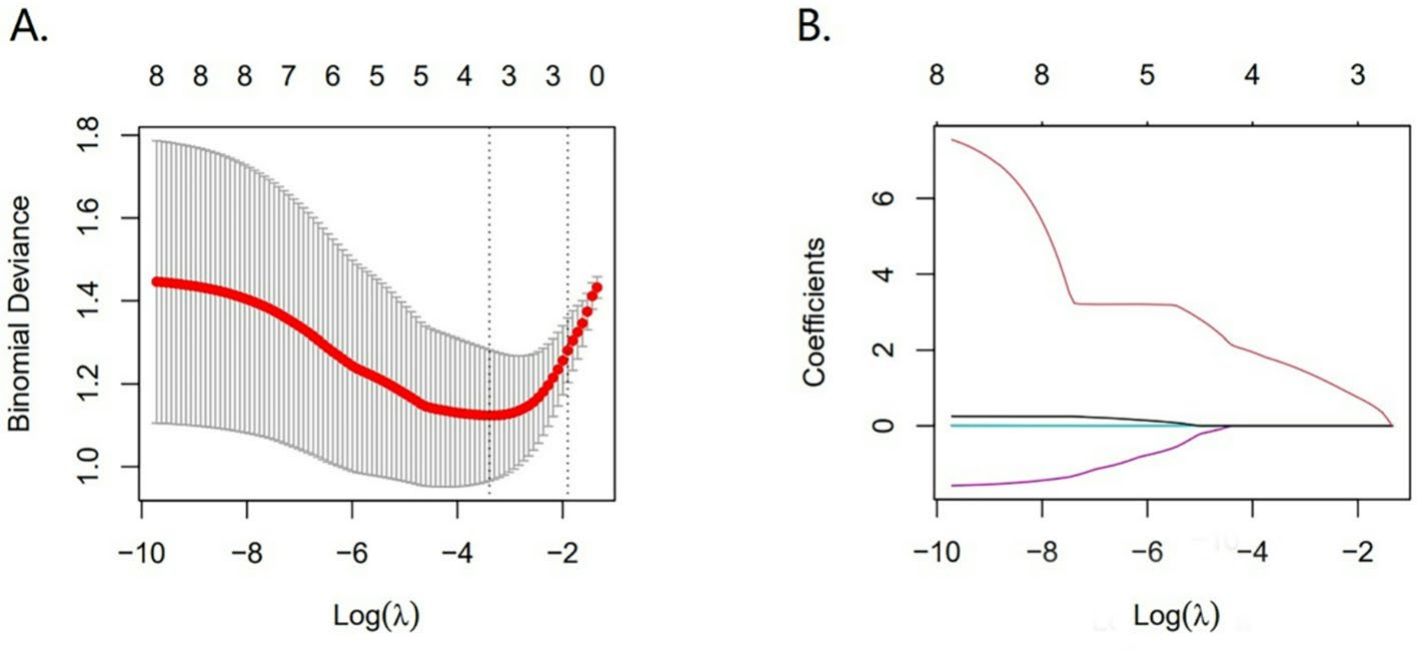
The result of LASSO analysis. A was the value of log(λ) and model error. Parameter (λ) selection was proceeded by 10-fold cross-validation via minimum λ in the LASSO model. B was LASSO coefficient profiles of the 5 baseline features

All the image parameters consisting of the meanADC, miniADC, maxiADC, medianADC, stdmeanADC, skewnessADC, kurtosisADC and Ratio-maxiADC/meanADC were evaluated by LASSO analysis preliminarily (Fig. 4). The result of LASSO analysis revealed that the kurtosisADC and Ratio-maxiADC/meanADC were eligible for FSLRM, SVM and RF models to differentiate subedema-rec and subedema-no-rec. The FSLRM revealed that Ratio-maxiADC/meanADC was the sole parameter that reached statistical significance in distinguishing between the subedema-rec and subedema-no-rec (*p* = 0.002, HR: 4.037) (Table 2). The best threshold value was 2.408 with sensitivity 79.2% and specificity 83.3%.

**Table 2:** The result of LASSO analysis and Stepwise logistic analysis

Image parameters	Result	*p* value	HR
	(LASSO analysis)	(logistic analysis)	
meanADC	Intercept	–	–
stdmeanADC	Intercept	–	–
maxiADC	Intercept	–	–
medianADC	Intercept	–	–
skewADC	Intercept	–	–
kurtADC	keep	0.277	-0.133
Ratio-maxiADC/meanADC	keep	0.002	4.037

**Fig. 5:**
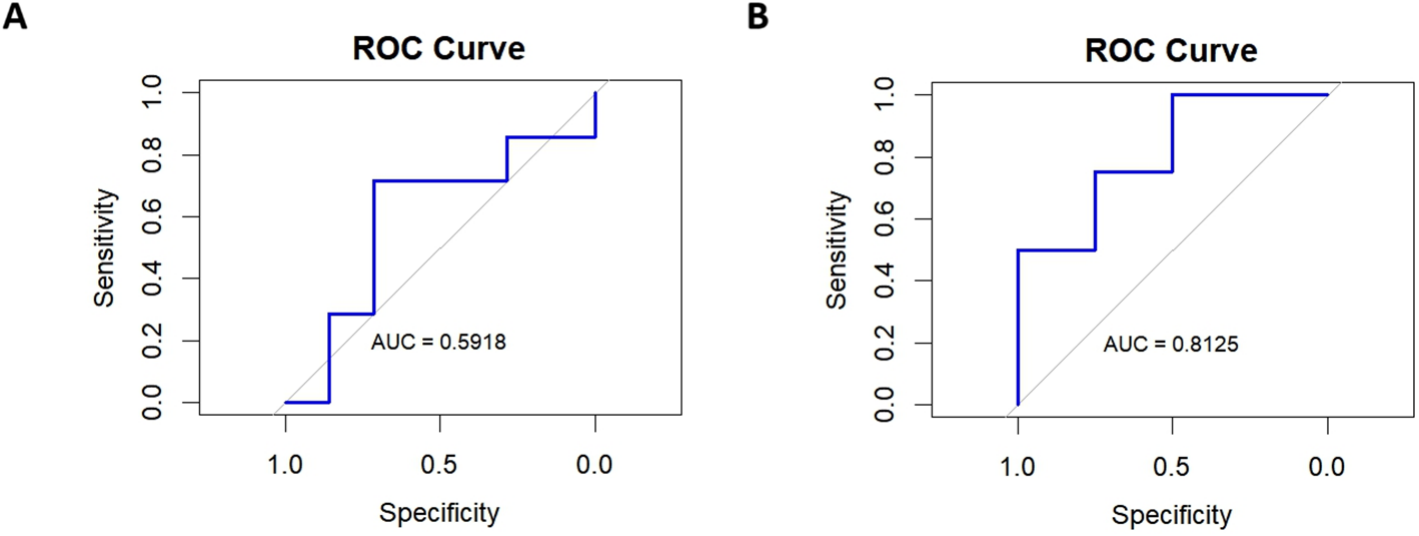
The ROC result graphs of the RF model and the SVM model. Figure A was the result of ROC curve of the RF model.and the AUC was 0.592.Figure B was the result of the ROC curve of the SVM model and the AUC value of this model was 0.813

**Fig. 6:**
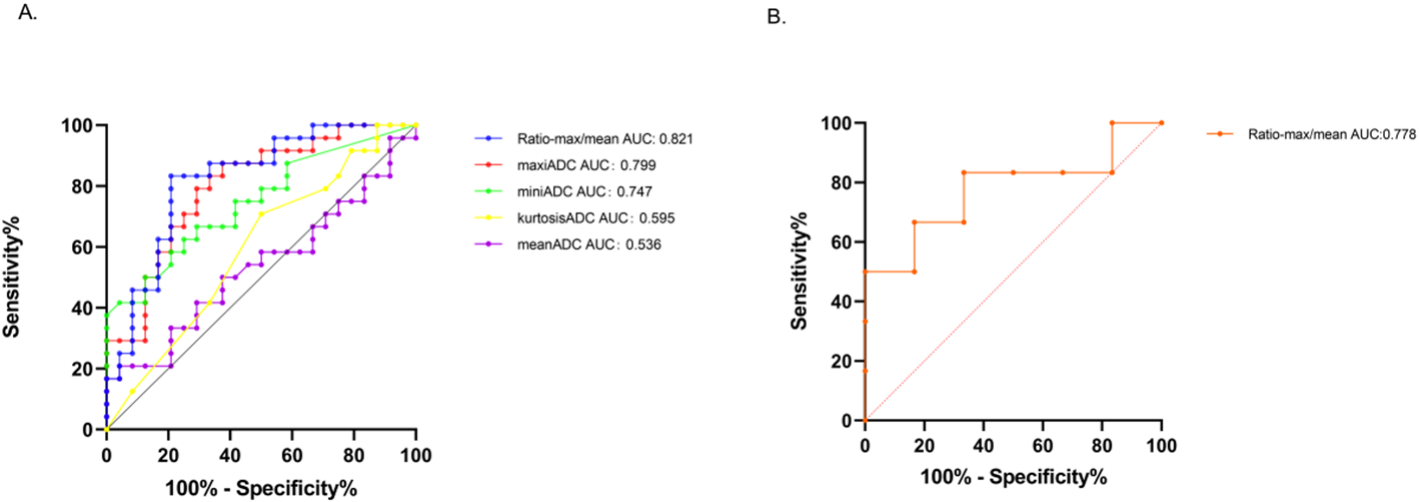
The AUC of models from parameters in the primary retrospective cohort and the validation retrospective cohort. (A) The AUC of the Ratio-maxiADC/meanADC was the best comparing to maxiADC, miniADC, meanADC and kurtosisADC. (B) The result of the AUC of the Ratio-maxiADC/meanADC in validation retrospective cohort

ROC analysis using the FSLRM demonstrated that the area under the curve (AUC) was 0.821. In contrast, the AUC values obtained via the SVM model and the RF model were 0.813 and 0.592(Fig. 5), respectively.In the retrospective validation cohort, the AUC of the Ratio-maxiADC/meanADC in differentiating the subedema-rec and subedema-no-rec was 0.778 (Fig. 6).

### Location prediction in image and pathological detection

**Fig. 7:**
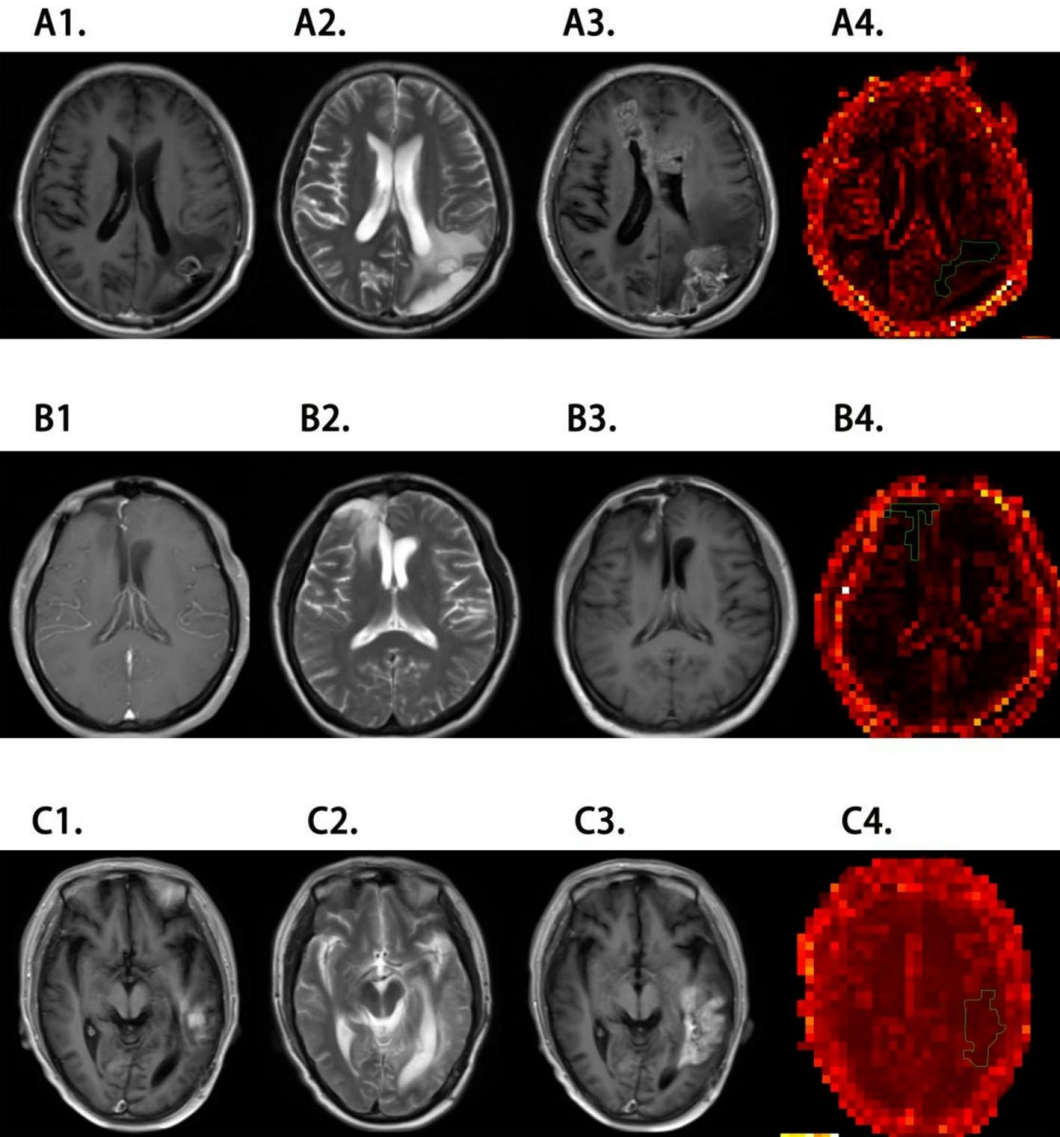
Location prediction of the subedema-rec on image of typical cases. A, a 63-year-old man. The lesion was located in the left occipital lobe. B, a 27-year-old woman. The lesion was located in the right frontal lobe. C, a 50-year-old man. The lesion was located in the left temporal lobe. The blue curve area in figures D1-3 was the high recurrence risk area within the predicted peritumoral edema area. A1, A2 and A3 were enhanced T1 sequences before tumor recurrence. B1, B2 and B3 were T2WI sequences before recurrence. C1, C2 and C3 were enhanced T1 sequences after recurrence. D1, D2 and D3 were prediction heat maps

Location prediction of subedema-rec by automatic image segmentation with unit of 2 × 2 voxel tiles was the best method to differentiate the subedema-rec and subedema-no-rec, comparing to other units of segmentation (Supplementary file 2). The predicted accuracy rate of tumor recurrent volume in the retrospective validation cohort ranged from 72.8 to 77.3% when using the segmentation unit of 2 × 2 voxel tiles, in contrast to other segmentation units. Specifically, the predicted accuracy rates of the segmentation units of 2.5 × 2.5 voxel tiles and 3 × 3 voxel tiles were approximately 60% and 50%, respectively. The result of typical patient was shown in Fig. 7.

**Fig. 8:**
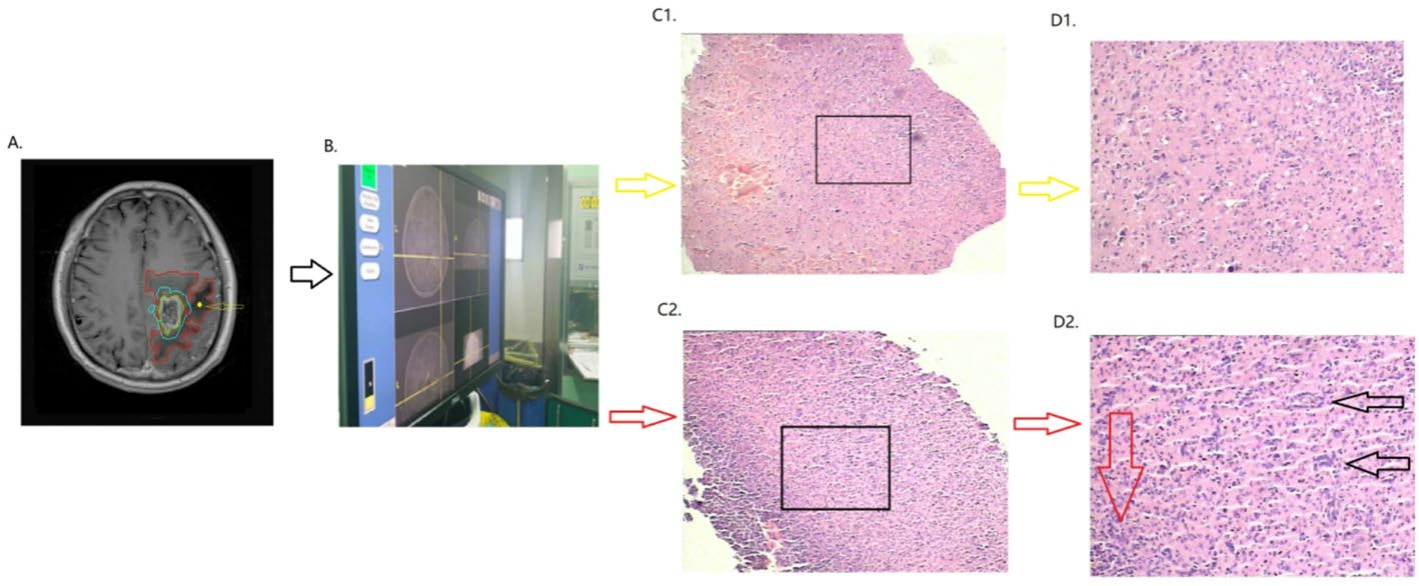
Pathology detection in typical case. (A) Enhanced T1 sequence before tumor recurrence merged by the boundary of tumor recurrence (green circle was the tumor lesion before tumor recurrence, blue circle was the tumor lesion after tumor recurrence, the red circle was the edema before tumor recurrence, the red point with the red arrow was the biopsy within subedema- rec and the yellow point with the yellow arrow was the biopsy within subedema-no-rec.). (B) The screenshot Surgical on biopsy guided by Navigation System. From C1 and C2 to D1 and D2. The pathological result of subedema- rec (red arrow) and subedema-no-rec (yellow arrow) were magnified into 10 × 10 (HE) (C1 and C2) and 10 × 40 (HE)(D1 and D2) from biopsy on the red point and yellow point

Pathological identification with multi-points biopsy was proceeded in 10 patients and the result revealed that tumor cell was dotted in subedema-rec while not in subedema-no-rec within peritumoral edema (Supplementary file 3). The typica patient was shown in Fig. 8.

## Discussion

Although it was controversy whether advanced MR techniques could succeeded in the investigation on the tumor micro-invasion, this study confirmed that the Ratio-maxiADC/meanADC on ADC map could identify subedema-rec [11–14]. Furthermore, this study proposed that the image prediction model basing on the Ratio-maxiADC/meanADC might be a feasible approach to predict the location of the subedema-rec before tumor recurrence, which was preliminarily explored in this study. The reason for the feasibility of this predicting model might be that functional MR could provide the information on the functional change in microenvironment in edema comparing to the conventional MR, which only could provide the information on anatomical change of peritumoral edema on image [15].

As the progress of tumor, the tumor microenvironment has changed. Although the anatomical structures such as neuronal cells and glial cells close to the tumor lesion have not undergone obvious changes, their functions have altered due to the direct or indirect influence of cytokines secreted by the tumor [16]. Research reported that tumor-associated macrophage-derived interleukin-1 played an important factor in the formation of the GBM-associated cerebral edema [17]. A similar situation may occur in subedema-rec. The physiological structure inside edema has not changed significantly, but the negative impact from tumor within subedema-rec on the activity of water molecules in the interstitial space of cells is affected. Some articles studying the mechanism of edema formation in GBM have reported the expression level change of molecules such as AQP4, which have an impact on the transport and the movement of water molecules in the interstitial space [18, 19]. Meanwhile, the changes of the activity of water molecules in certain regions can be detected by functional sequences such as DWI, DTI, PWI and so on, which is also a research hotspot of the clinical application of functional MR on the tumor microenvironment in recent years. Research proposed a voxel - wise deep learning - based peritumoral microenvironment index. This index was based on the DTI and aimed to predict the extent of tumor cell infiltration within peritumoral edema in GBM. However, the efficiency of this method was not reported [20]. MRI/histology voxel-to-voxel comparison revealed a negative correlation between tumor cell infiltration and perfusion at the tumor margins by research, but the location prediction of tumor cell infiltration inside peritumoral edema was not presented using perfusion parameter in that research [21].

Study tied to compare MRS and ^18^F-dihydroxyphenylalanine (DOPA) PET in investigating infiltrative gliomas, and only the ^18^F-DOPA uptake was an statistically confimed image parameter [22]. In the meantime, researchers had also explored ASL and DTI or combined DTI, DKI with PWI to achieve a better AUC in the investigation of tumor microinvasion in peritumoral edema [23]. Nevertheless, none of them were employed to assess the subedema-rec. Overall, the functional change usually happened before anatomical change in tumor microenvironment which might be the reason that the functional MR had more promise chance in successively predicting the subedema-rec in GBM effectively than conventional MR.

Given the potential of functional MRI in predicting subedema - rec, it is essential to further explore the specific imaging parameters that can accurately reflect the tumor microinvasion within the peritumoral edema. Recent research explored to evaluate tumor infiltration using the histogram character of ADC map. Study had proved that the rADC (The ratio between mean ADC value of contrast-enhancing lesion and the matching structure in the contra-lateral hemisphere) in DWI image could give indication on the molecular type in glioma [24]. But the method of applying rADC on identifying the subedema-rec was infeasible because the difference between mean ADC of the subedema-rec and subedema-no-rec was not significant different. Although miniADC was considered as a potential factor to represent tumor cellularity in tumor lesion, research revealed that miniADC could not efficiently reflect tumor microinvasion within edema [25–29]. As the above published revealed that the miniADC of edema was influenced by tumor, necrosis, image noise and other unidentified factors. As a comparison, the meanADC of edema was a relatively stable factor reflecting the general situation of ROI so that it might be suitable for being used as a control factor. It was demonstrated that the maxiADC inside edema was significantly different between subedema-rec and subedema-no-rec by this study. This phenomenon might reflect that the tumor microinvasion in the edema leaded to the extreme changes of the activity capacity of water molecules, which the activity capacity inside subedema-rec was limited in general while the activity capacity of a small part inside this region increased significantly on the contrary. No matter what, the disorder of the activity capacity of water molecules inside subedema-re was the result of impairment on transport regulation ability of water molecular in microenvironment. This complicated situation implied that sole parameter from functional MR image could not correctively reflect the change of the activity capacity of water molecules in peritumoral edema. The Ratio-maxiADC/meanADC consisting of the information of maxiADC and meanADC proposed by this study had advantages over a single image parameter to explore the complicated situation of the microenvironment of peritumoral edema. In addition, this study demonstrated that meanADC and miniADC of tumors decreased, while maxiADC increased with tumor recurrence, consistent with published research [30].

In this study, a method of establishing a prediction model was employed, which involved using the LASSO analysis to screen parameters and combining it with the FSLRM for comparison with machine learning algorithms. It was found that, overall, the performance of machine learning algorithms was inferior to that of the combination of the LASSO analysis and FSLRM. This suggests that when establishing a model using a single imaging sequence, a linear correlation model after removing collinearity may be simpler and more stable.

This is consistent with the reported research findings [31, 32]. After removing the collinearity factors from the research subjects, the inherent stable linear correlation can be effectively excavated. This has better interpretability and generalizability compared with the machine learning algorithm model.

Although this study revealed the the Ratio-maxiADC/meanADC on ADC map could indicate the sub volume of edema with high risk for tumor recurrence, this study still had several limitations. First, this study contained retrospective research cohort and confounding factors could not be avoided, but this study consisted a small-scale retrospective validation cohort to evaluate the applicational value of the result of this study. Second, although the point-to-point identification between MR image and pathological detection were proceeded in 10 patients, beside typical tumor cell infiltration identified, atypical tumor cell infiltration was observed within the subedema-rec in part of cases. This phenomenon might be related to the pathogenesis process of the formation of subedema-rec. The phenomenon of atypical tumor infiltration might be a transitional stage of typical tumor infiltration. At the stage of atypical tumor infiltration, the microenvironment of subedema-rec had already changed. However, the molecular mechanism of microenvironmental changes in subedema-rec was not investigated because it went beyond the main objective set by this study. But subsequent research has been included in the next research plan. Third, location prediction using automatic image segmentation were only explored using several units in this study, which the best units used for location prediction of the subedema-rec would be missed. But this study was pilot research that proposed a reasonable method to predict the subedema-rec. Further research plan based this preliminary result would explore the most suitable automatic segmentation unit.

In summary, this study offered a simple and promising approach comprising the maximum ADC value and the mean ADC value to explore the subvolume with a high likelihood of tumor recurrence within peritumoral edema, which might provide a hint in evaluating surgical margins or postoperative radiotherapy target boundary.

## Electronic supplementary material

Below is the link to the electronic supplementary material.

Supplementary Material 1

## Data Availability

The data that support the findings of this study are not openly available due to patient privacy and ethical considerations. These data contain sensitive patient - related information, including medical records and imaging data. However, the data are available from the corresponding author, Baosheng Li, upon reasonable request. The data are securely stored in the controlled access data storage of Linyi People’s Hospital affiliated with Shandong Second Medical University. To obtain access, researchers should submit a detailed request to the corresponding author, clearly stating the research purpose, data usage plan, and ensuring compliance with relevant ethical and legal requirements.
