# Supplementary material for: Pilot research on predicting the sub-volume with high risk of tumor recurrence inside peritumoral edema using the ratio-maxiADC/meanADC from the advanced MRI
Source: Discov Oncol. 2025 Sep 24;16:1697. doi: 10.1007/s12672-025-03199-3 (PMC12460852; doi:10.1007/s12672-025-03199-3)
Supplement: Supplementary file 1 — Supplementary Material 1 [file 12672_2025_3199_MOESM1_ESM.docx]

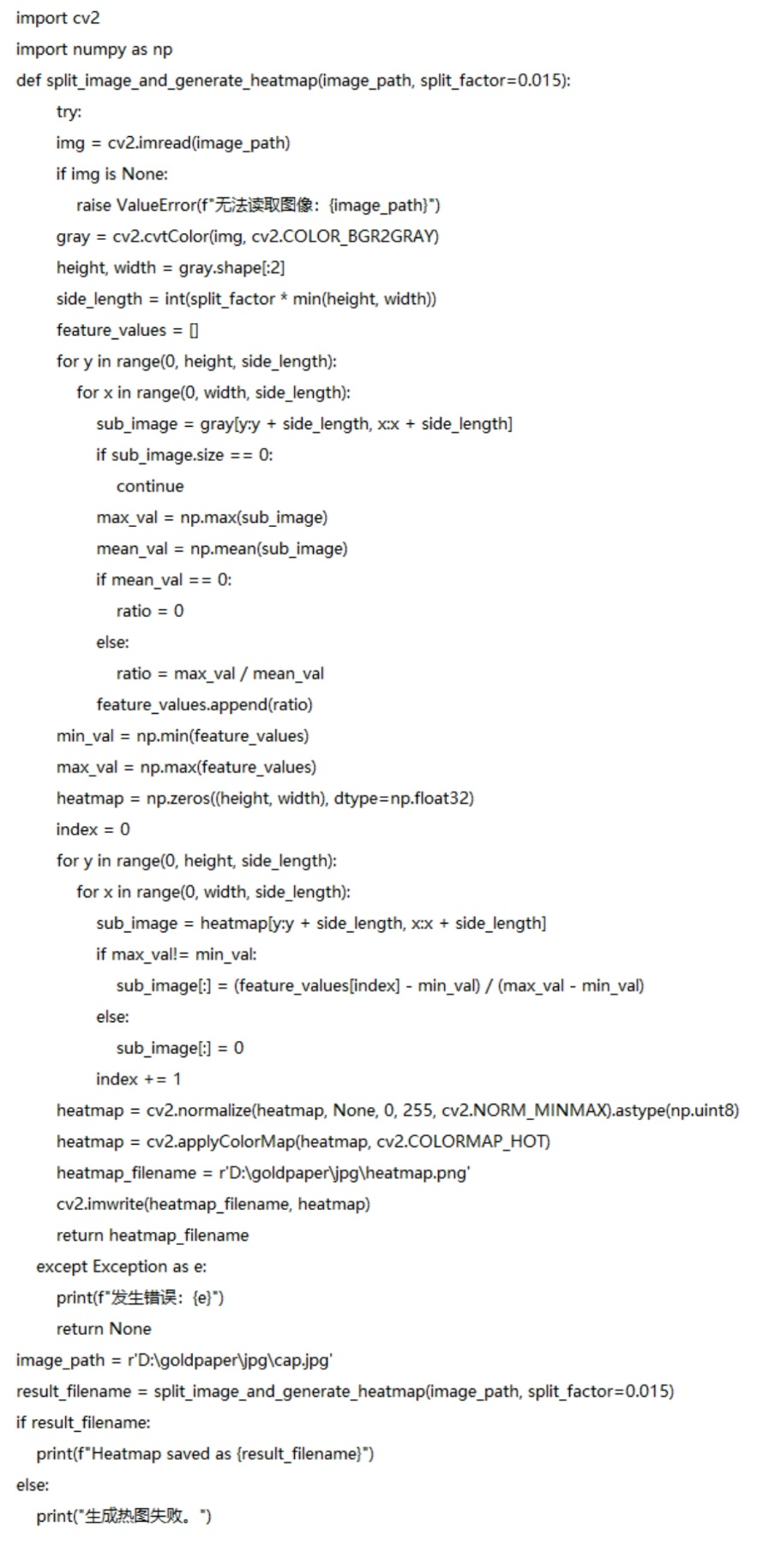


**Supplement file 1:** command code


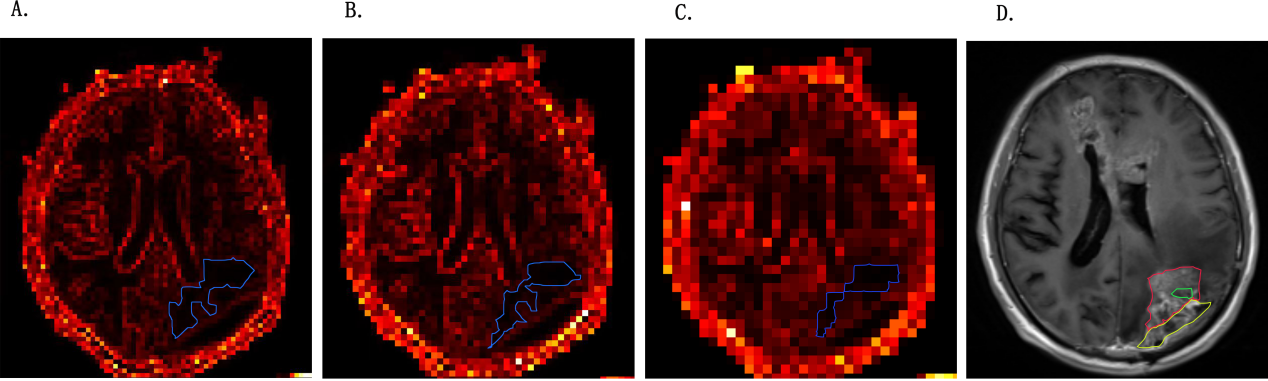


**Supplementary file 2.**

The heat map produced by auto segmentation of different units.

Segmentation units compromised of 2 × 2 voxel tiles (A), 2.5 × 2.5 voxel tiles (B) and 3 × 3 voxel tiles (C). D: enhanced T1 sequence. Yellow curve: surgical cavity, green curve: tumor range before recurrence. Red curve: tumor range after recurrence.


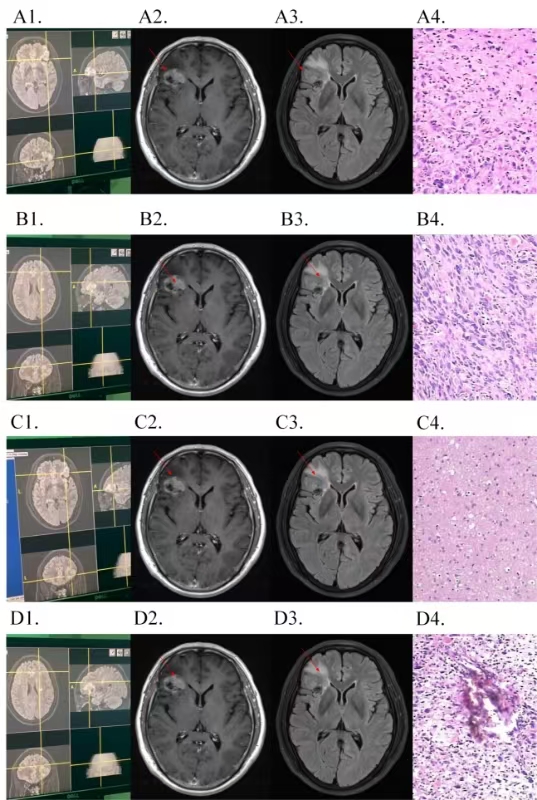


**Supplement file 3.**

The typical case of point-to-point comparison of images and cases, multi-point puncture and pathological examination.

Points A, B, C and D are multiple pathological sampling points for the same case. A1, B1, C1 and D1 were preoperative surgical navigation maps. The intersection of the horizontal and vertical lines was the planned biopsy position. A2, B2, C2 and D2 were preoperative enhanced T1 - weighted magnetic resonance sequences, and the red arrows indicated the planned biopsy sampling points. A3, B3, C3 and D3 were preoperative T2 - weighted magnetic resonance sequences, and the red arrows indicated the planned biopsy sampling points. A4, B4, C4 and D4 were the results of HE staining in pathological examination.
